# Effects of Offset Pixel Aperture Width on the Performances of Monochrome CMOS Image Sensors for Depth Extraction

**DOI:** 10.3390/s19081823

**Published:** 2019-04-16

**Authors:** Jimin Lee, Byoung-Soo Choi, Sang-Hwan Kim, Jewon Lee, Junwoo Lee, Seunghyuk Chang, JongHo Park, Sang-Jin Lee, Jang-Kyoo Shin

**Affiliations:** 1 School of Electronics Engineering, Kyungpook National University, 80 Deahak-ro, Buk-gu, Daegu 41566, Korea; jmLee@ee.knu.ac.kr (J.L.); bschoi@ee.knu.ac.kr (B.-S.C.); shkim7@knu.ac.kr (S.-H.K.); jewonlee@knu.ac.kr (J.L.); junwoolee@knu.ac.kr (J.L.); 2 Center for Integrated Smart Sensors, KAIST, 291 Daehak-ro, Yuseong-gu, Daejeon 34141, Korea; schang71@kaist.ac.kr (S.C.); parkjh20@kaist.ac.kr (J.P.); sjlee82@kaist.ac.kr (S.-J.L.)

**Keywords:** offset pixel aperture width, monochrome, CMOS image sensor, depth extraction

## Abstract

This paper presents the effects of offset pixel aperture width on the performance of monochrome (MONO) CMOS image sensors (CISs) for a three-dimensional image sensor. Using a technique to integrate the offset pixel aperture (OPA) inside each pixel, the depth information can be acquired using a disparity from OPA patterns. The OPA is classified into two pattern types: Left-offset pixel aperture (LOPA) and right-offset pixel aperture (ROPA). These OPAs are divided into odd and even rows and integrated in a pixel array. To analyze the correlation between the OPA width and the sensor characteristics, experiments were conducted by configuring the test elements group (TEG) regions. The OPA width of the TEG region for the measurement varied in the range of 0.3–0.5 μm. As the aperture width decreased, the disparity of the image increased, while the sensitivity decreased. It is possible to acquire depth information by the disparity obtained from the proposed MONO CIS using the OPA technique without an external light source. Therefore, the proposed MONO CIS with OPA could easily be applied to miniaturized devices. The proposed MONO CIS was designed and manufactured using the 0.11 μm CIS process.

## 1. Introduction

An image is an important method by which visual information is shared in modern society. However, it is difficult to obtain stereoscopic information using images reproduced by general image sensors. Human eyes can sense distance by accepting different visual information separately. However, distance information is not included in general camera systems due to their chips having only a single image sensor. Many studies have focused on realizing better stereoscopic images to obtain distance information. In 1870, David Brewster invented the Brewster stereoscope using the binocular parallax [1]. Subsequent research started realizing stereo vision in earnest. In other words, the acquisition of depth information is indispensable to producing stereoscopic images. In addition, depth information can both reproduce stereoscopic images and could be utilized in various fields, such as vehicle smart sensors, augmented reality, and indoor mapping [2,3,4].

Many researchers remain immersed in the development of three-dimensional (3D) imaging systems such as time-of-flight (TOF), light field, and stereo vision. Stereo vision is a method of creating stereoscopic images by combining each image acquired using two cameras [5,6,7,8,9]. The distance is calculated based on triangulation by finding the corresponding point of two cameras. TOF technology is a technique that emits an optical signal from an interlocked infrared source with a camera, measures the time for the emitted light to be reflected by the object and return, and thus obtains the depth information [10,11,12,13,14]. Therefore, an infrared source with high power consumption is necessary for the TOF system. The light field technique contains microlens arrays that are integrated at a small distance from the image sensor [15,16,17,18,19]. Microlens arrays are designed in positions in which the object’s image is focused on the camera lens. The light transferred from the object passes first passes through the camera lens. Simultaneously, the location on the camera lens through which the light passes is different depending on the object’s position. In other words, the output of the sensor due to the light passing through the microlens is proportional to the amount of light that corresponds to the location on the lens of the camera through which the light passes. Therefore, because the light field image sensor contains as many sets of disparity information as the number of microlenses, a stereoscopic image can be reproduced.

Recently, the miniaturization of portable devices, such as mobile phones and tablet PCs, in which CMOS image sensors (CISs) are integrated, has been performed with advancements in technology. This is due to advantages such as their small required area, easy portability, and low power consumption. Some of the disadvantages of the above 3D imaging system technology include the need for an infrared source that has high power consumption, the use of a plurality of cameras, and the sensor’s limited resolution depending on the number of microlenses. However, the proposed monochrome (MONO) CIS with offset pixel apertures (OPAs) is advantageous to acquire depth information using only one sensor and requires no infrared source. Furthermore, concerns regarding the loss of resolution caused by the number of microlenses, which serve as the light field system, is nonexistent.

This paper is organized as follows. The sensor’s architecture is described in Section 2. The measurement of the disparity depending on the distance and measurement of the chief ray angle (CRA) with OPA is described in Section 3. Finally, Section 4 contains the conclusions.

## 2. Architecture of the Sensor

### 2.1. Pixel Structure

**Figure 1:**
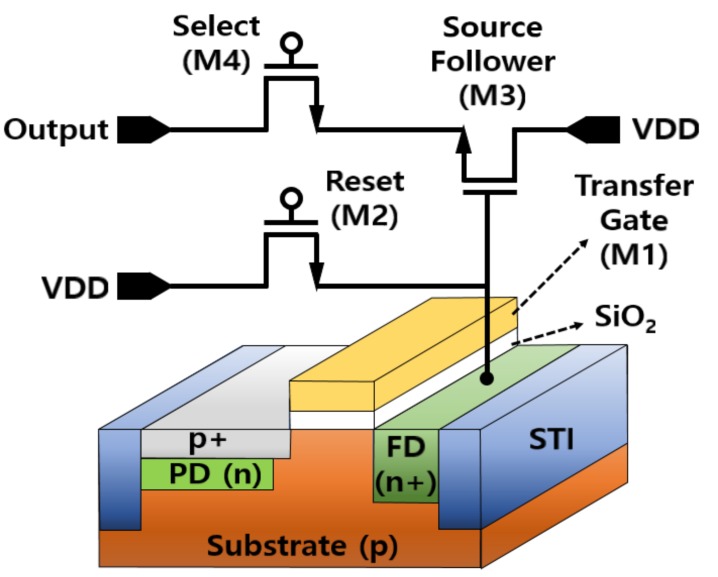
Cross-section of the conventional four-transistor (4-Tr) active pixel sensor (APS) structure.

This paper proposes obtaining depth information using a MONO CIS with OPA. The pixel of the proposed MONO CIS with OPA forms the conventional four-transistor (4-Tr) active pixel sensor (APS) as shown in Figure 1. Since the pixel of the proposed MONO CIS with OPA was based on the front side illumination (FSI) method, a pinned photodiode (PPD) structure is applied to reduce the defects generated on the surface of the photodiode (PD). The 4-Tr APS is composed of a transfer gate transistor (M1), a reset transistor (M2), a source follower (M3), and selection transistors (M4). The transistors (M1–4) are integrated into a single pixel. The operation principle of a pixel with the 4-Tr structure is described as follows: When the light is incident on the pixel, signal electrons are generated from the PD, and are then transferred to the floating diffusion (FD) node using M1. The signal electrons transferred from the PD are transmitted to M3.

When M4 is turned on, the signal electrons that pass through M3 are transferred to the readout circuit. The role of the M2 is to remove residual signal electrons from the FD node after transferring the signal electrons. In the proposed MONO CIS, the 4-Tr APS structure was applied to improve the sensitivity and conversion gain of the pixel. Moreover, to prevent crosstalk caused by the surrounding pixels, the pixel was isolated using the shallow trench isolation (STI) process, which was used to isolate a device by forming a trench in a semiconductor substrate and filling it with silicon oxide.

The proposed MONO CIS was designed using the 0.11 μm CIS process. The size of the pixel was 2.8 × 2.8 μm and the measurement was performed. The pixel array of the proposed MONO CIS with OPA was designed without color filters and the OPA was integrated at the pixel level.

### 2.2. MONO CIS with OPA Technique

**Figure 2:**
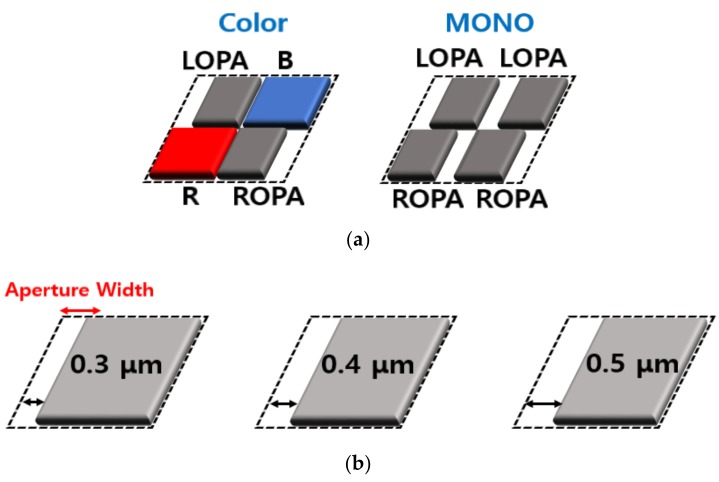
(**a**) Comparison of offset pixel aperture (OPA) patterns between the color and the monochrome (MONO) CMOS image sensor (CIS), using the OPA technique; (**b**) the variation of OPA width for measuring the performance difference.

The OPAs of the MONO CIS were designed using the first metal layer to create a rectangular opening area at only a certain position of the PD [20]. The disparity of images was determined by the width and position of the opening in the OPA. Figure 2a shows a comparison of the OPA patterns between the color and the MONO CIS using the OPA technique. A previously studied CIS with OPA had a color filter array (CFA) [21,22], the pattern of which was composed of the left-offset pixel aperture (LOPA), blue (B), red (R), and the right-offset pixel aperture (ROPA). The LOPA and ROPA were diagonally located in the pattern and the CIS had a one-pixel difference in the disparity, although the camera was focused. The MONO pattern was comprised of two pattern types, including just the LOPA and ROPA. The two types of pattern were divided into odd and even rows and were integrated into a pixel array. The proposed MONO CIS was fabricated without the color filters and the height of the pixel was low because the CFA was not necessary. This is an advantage of being able to improve the disparity due to the lower pixel height. The MONO pattern was formed in whole pixel arrays. Figure 2b shows the variation in the OPA width for measuring the performance difference. The width of the opening area in the OPA was in the range of 0.3–0.5 μm, and the extent of the offset was designed to have a distance in the range of 1.15–1.25 μm. The offset is defined as the distance between the center of the pixel and the center of the OPA.

**Figure 3:**
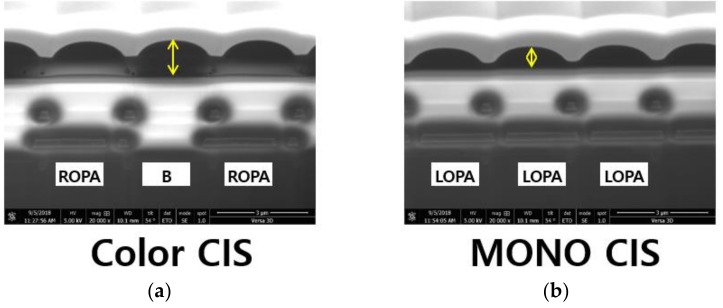
(**a**) Pixel height of the color CIS; (**b**) pixel height of the proposed MONO CIS.

Figure 3 shows the difference in the pixel height between the color CIS and the proposed MONO CIS. To confirm the difference in pixel height, a cross-section of the actual fabricated chip was taken using the focused ion beam (FIB) system. As shown in this figure, the proposed MONO CIS was made with the exception of the color filter process. The pixel height of the proposed MONO CIS was lower than the color CIS, which is the same as increasing the focal length. Therefore, the disparity was improved compared to the color CIS [23].

## 3. Results and Discussions

### 3.1. Measurement of the Disparity Depending on the Distance

In the proposed MONO CIS with OPA, the disparity was as effective as the binocular parallax. Because the two types of OPA were designed to yield a constant offset in the symmetric direction, the difference in the disparity changed according to the distance between the object and the sensor. Subsequently, the difference in the disparity was proportional to the distance.

**Figure 4:**
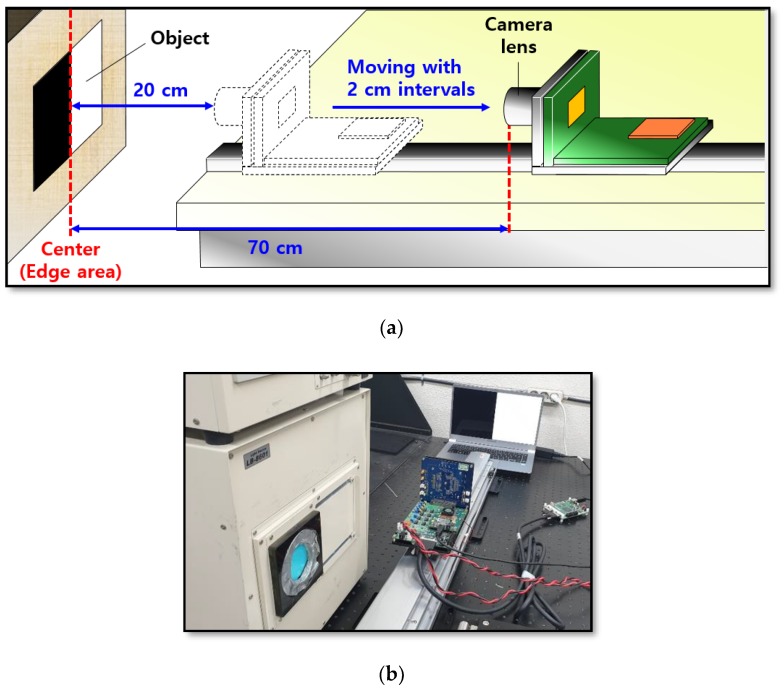
(**a**) Measurement environment for the evaluation of the disparity; (**b**) photograph of the actual system for measuring the disparity depending on the distance.

In this study, we measured the disparity according to the distance using the proposed MONO CIS with OPA. Figure 4a shows the measurement environment for the evaluation of disparity. The object is the black-and-white pattern image for the measurement. Using the boundary between black and white renders it easier to compare disparities in both types of OPA. First, a black-and-white pattern was displayed using a laptop computer. The reason for using a laptop computer was that the edge portion of the black and white pattern could be more clearly distinguished by controlling the monitor’s brightness. Next, the camera lens of the proposed MONO CIS with OPA was placed 20 cm from the object. Here, it was important to place the edge area of the black-and-white pattern at the center of the sensor. In this measurement, we applied a method in which a line at the center of the pixel array was marked on the display area of the image sensor driving the program, and this was matched with the edge area of the object. Subsequently, the measurement proceeded by moving the proposed sensor at 2 cm intervals from 20 cm to 70 cm, using a guide rail. Figure 4b is a picture of the actual measurement system for determining the disparity depending on the distance.

**Figure 5:**
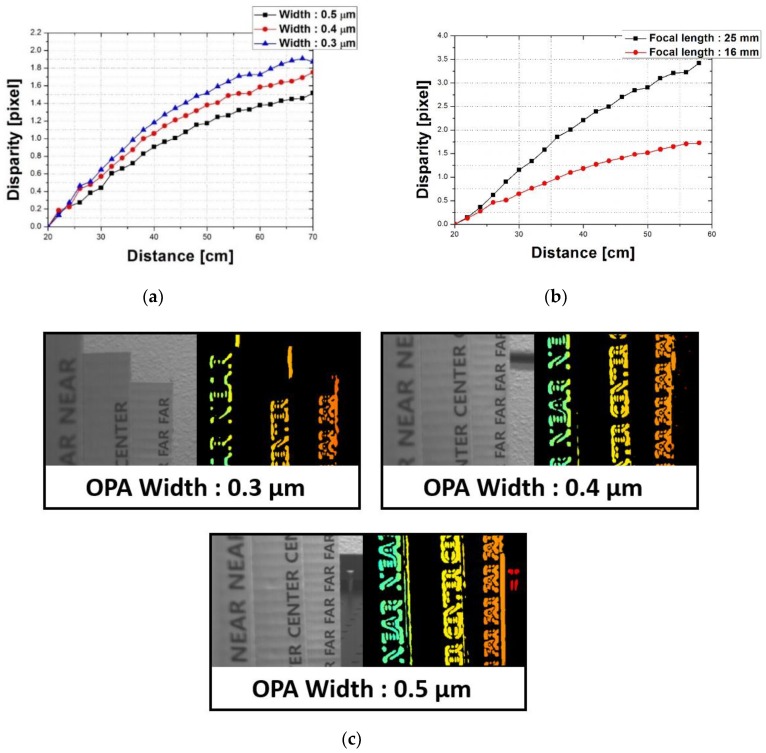
(**a**) Measurement results for the disparity difference depending on the OPA width and distance; (**b**) change in the disparity when using a camera lens with a 25 mm focal length and 0.3 μm width of OPA; (**c**) images of the depth map depending on the OPA width.

Figure 5a shows the measurement results of the disparity difference depending on the OPA width and distance. The method for calculating the disparity was as follows. The channel data of LOPA and ROPA were separated from 100 frames of the measurement images using the proposed MONO CIS with OPA, and each channel data were averaged. Hence, it was possible to confirm the image of the edge area for each of the two divided types of OPA. The degree of disparity was calculated by subtracting the difference between the location of the edge from the LOPA image and that from the ROPA image. As shown in Figure 5a, the disparity increased as the distance between the sensor and the object increased. The distance could be inferred according to the degree of disparity. In other words, the degree of depth can be understood using the variation in the disparity. In addition, the degree of offset that varies depending on the OPA width affects the disparity. As the degree of offset increased, the disparity between the LOPA and ROPA increased. Therefore, the disparity was largest when the OPA width was 0.3 μm. The disparity was only about two pixels at the maximum when the OPA width was 0.3 μm because we used a camera lens with a small focal length. In this experiment, a camera lens with a 16 mm focal length was used. The reason for using a small focal length camera lens was to ensure a wide field-of-view (FOV) range. The disparity was dependent on the degree of focal length and increased when using a camera lens with a large focal length. Figure 5b shows the change in the disparity when using a camera lens with a 25 mm focal length and 0.3 μm width of OPA. As shown in Figure 5b, using a camera lens with a large focal length increased the disparity. Figure 5c shows images of the depth map depending on the OPA width. The object used was black characters printed on white paper so that the edge information could be clearly measured using the definite intensity difference between black and white. The distance between the nearest object and the camera lens was 30 cm and the interval between objects was 10 cm. The camera lens was focused on the object farthest away. Focusing on close object increased the degree to which distant objects blurred. As the degree of blur increased, the depth information became inaccurate due to the disparity. Therefore, the camera lens was focused on the object farthest away so that the disparity depth map could be implemented more accurately.

### 3.2. Characteristics of the Proposed MONO CIS with OPA Depending on the CRA

This section describes the measurement method and results regarding the characteristics of the proposed MONO CIS with OPA depending on the CRA described. As confirmed by the measurement results in the previous section, the disparity in the proposed sensor was generated by the LOPA and ROPA. The measurement method for changing the angle of the chief ray was used to confirm the degree of disparity. The chief ray is the ray that passes through the center of the camera aperture. The influence of the LOPA and ROPA from the pixel output is confirmed by the result of the sensor according to the angle of the chief ray using this measurement method.

**Figure 6:**
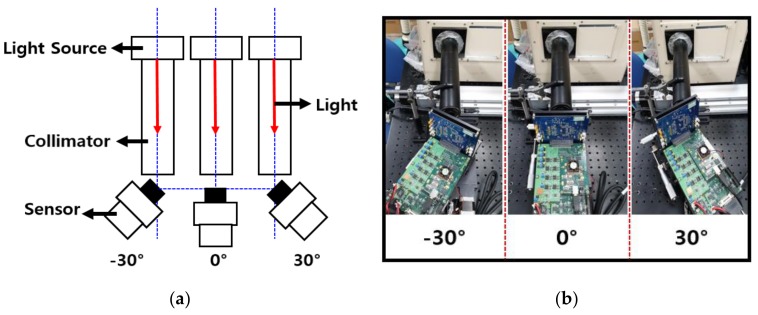
(**a**) Measurement environment for the performance of the CIS depending on the chief ray angle (CRA); (**b**) photographs of the actual measurement system for CRA analysis.

Figure 6a shows the measurement environment for the performance of the CIS depending on the CRA, and Figure 6b shows photographs of the actual measurement system for CRA analysis. The collimator is connected to the light source and the length of the connected collimator is 282.6 mm. The collimator must always be connected horizontally; the angle of the incident light is a crucial factor in the measurement, so even a slight deviation will significantly influence the measurement result. When changing the angle of the sensor, the collimator and sensor were placed far apart to prevent the range limitation of the sensor angle changing with the collimator. The camera lens was removed in the measurement, and the light from the light source was directly transferred to the sensor. The centers of the collimator and the sensor match were set to the front area of the sensor, and it was ensured that the central axis of the sensor did not shift. In addition, they were fitted such that the angle of the sensor could be adjusted around the center of the sensor axis. In the measurement system, the light’s incidence angle could be changed by controlling the angle of the sensor. A rotary machine was used to control the sensor angle; this machine is a worm gear type and is controlled using a step motor; when the input is a constant pulse, the angle of the sensor changes correspondingly. The reason for using the rotary machine was to improve the accuracy of the angle change. The outputs of the MONO CIS with OPA depending on the angle of the sensor were measured at 2° intervals from −30° to 30°.

**Figure 7:**
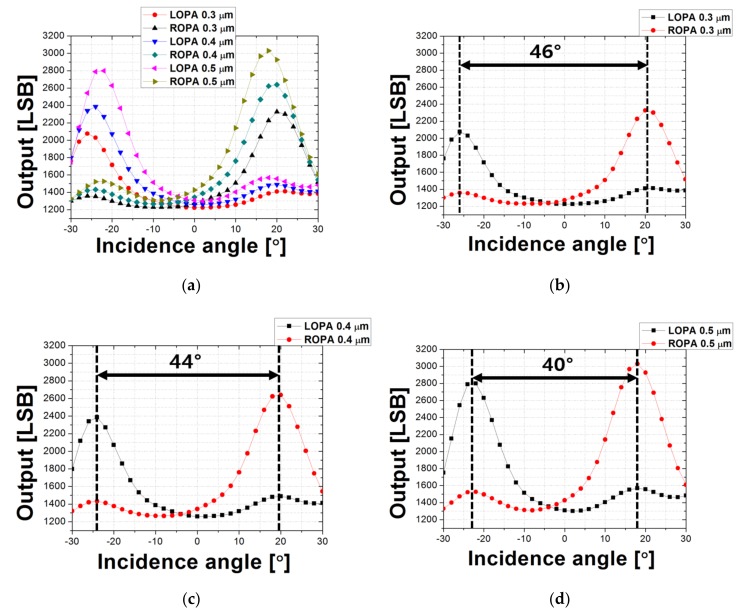
(**a**) Measurement results of CRA depending on the OPA width; (**b**) disparity of MONO CIS with 0.3 μm OPA width; (**c**) disparity of MONO CIS with 0.4 μm OPA width; (**d**) disparity of MONO CIS with 0.5 μm OPA width.

**Figure 8:**
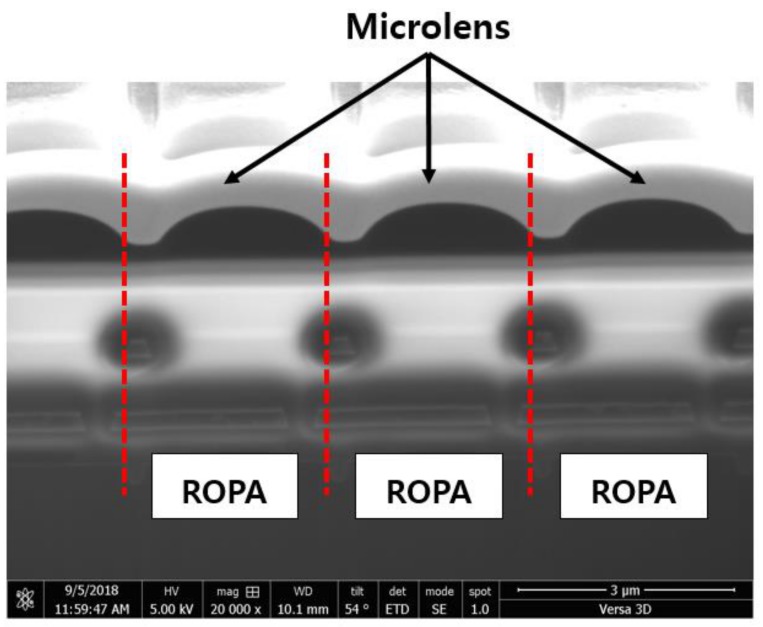
Image obtained by photographing the position of the microlens shifted using the focused ion beam (FIB) system.

Figure 7a shows the measurement results for CRA depending on the OPA width. As shown in Figure 7a, the origin was shifted closer to −5° than 0° because the location of the microlens in the pixel had shifted slightly. However, the origin shifting error was not a critical issue in the CRA measurement results because the purpose of the measurement was to analyze the difference in the incidence angle from the LOPA and ROPA channels with the maximum output. Figure 7b–d show the results of analyzing the degrees of disparity depending on the width of the OPA. The disparity was 46° when the OPA width was 0.3 μm and was 44° when the OPA width was 0.4 μm. In addition, the proposed MONO CIS with 0.5 μm OPA width had 40° disparity. As the width of the OPA increased, the degree of disparity decreased, as shown in this result. However, when the OPA width decreased, the optical power that was transmitted to the photodiode decreased. In other words, the sensitivity increased as the width of the OPA increased. As shown in Figure 7a, an output difference occurred between the LOPA and ROPA in the measurement result. Figure 8 shows the image obtained by photographing the position of the microlens shifted using the FIB system. When designing a pixel, the center of the microlens was designed to coincide with the center of the photodiode area. However, fabrication process problems meant that the position of the microlens had shifted slightly to the right and had been integrated. This caused a difference in the amount of light transmitted from the microlens to the photodiode through the aperture depending on the incidence angle. Therefore, the output of the LOPA was lower than that of the ROPA. Table 1 summarizes the proposed MONO CIS with OPA.

## 4. Conclusions

In this study, we studied the effects of offset pixel aperture width on the performances of monochrome CMOS image sensors for depth extraction. The proposed MONO CIS with OPAs was designed and manufactured using a 0.11 µm CMOS process. The pixel array of the proposed MONO CIS with OPA was designed without color filters to produce a monochrome image. The OPA was designed using the first metal layer in the CIS process. In addition, the MONO pattern was composed of LOPA and ROPA pixels. The LOPA and ROPA were arranged on odd and even rows across the whole pixel array area.

In the experimental results, the degree of disparity increased as the distance increased between the camera system and the object. Further, because the two respective types of OPA exhibited different CRA characteristics, there was a difference in the disparity. Therefore, adjusting the position and width of the opening area enabled adjusting the difference in the CRA when designing the OPA pattern. Depth information could be acquired using the disparity that occurred in the pixel without requiring external factors, such as a high-power infrared source, plurality of cameras, etc. Therefore, this could be applied to the imaging device side that requires miniaturization, and stereoscopic images could be utilized.

## Figures and Tables

**Table 1:** Summary of the proposed MONO CIS with OPA.

	Width 0.3 μm	Width 0.4 μm	Width 0.5 μm
Pixel size	2.8 × 2.8 μm		
Pixel array	486 × 1080 (test element group (TEG) region only)		
Frame rate	18 fps		
Process	0.11 μm CIS process		
Color pattern	Monochrome (without color filters)		
Pixel type	4-Tr APS structure		
Chip size	7 mm (H) × 10 mm (V)		
Power	Analog: 3.3 V. Digital: 1.8 V		
Disparity	46°	44°	40°
